# Editorial: Natural killer cell plasticity and diversity in antiviral immunity

**DOI:** 10.3389/fimmu.2023.1175111

**Published:** 2023-03-14

**Authors:** Philippe Krebs, Hui Peng, Vikas Duhan

**Affiliations:** ^1^ Institute of Tissue Medicine and Pathology, University of Bern, Bern, Switzerland; ^2^ Institute of Immunology, University of Science and Technology of China, Hefei, Anhui, China; ^3^ Tumor Immunology Laboratory, QIMR Berghofer Medical Research Institute, Herston, QLD, Australia

**Keywords:** NK cell, viral infection, immunity, plasticity, diversity

## Summary

Natural killer (NK) cells are a type of cytotoxic innate immune cells that recognize and kill virus-infected cells and cancer cells. NK cells are composed of multiple subsets, each with a high degree of diversity based on their expression of various inhibitory-activating receptors, chemokine receptors and transcriptional factors. During virus infection, exposure of NK cells to different cytokines and interaction with infected cells promote alteration in their NK cell receptor repertoire and differentiation into a new or merge with an existing subset, demonstrating their plastic nature. Such NK cell plasticity can have a significant impact on NK cell phenotypic and functional diversity, potentially promoting antiviral immunity or resulting in virus immune evasion, ultimately influencing disease outcomes. In this Research Topic, we present and discuss the latest research on NK cell plasticity and diversity in viral infections, as well as its therapeutic implications.

## NK cell diversity

NK cells were previously considered a homogeneous population of lymphocytes with limited diversity and fixed phenotypic and functional properties in both humans and mice. The peripheral blood of healthy patients comprises a main subset of cytotoxic CD56^Dim^ cells and a smaller fraction of CD56^Bright^ cells with limited cytotoxicity (Abel et al.). However, recent developments in NK cell biology have revealed a higher diversity of NK cells based on receptor repertoire, transcription factors, tissue distribution, and functionality, which indicate multiple NK cell subsets (1, 2). NK cell diversity depends on several factors, including host genetic composition such as mutations in killer-cell immunoglobulin-like receptors (KIRs) and human leukocyte antigens (HLA) genes, epigenetic regulators, interactions with other cell types, and environmental factors such as viral infections (3, 4). The degree of diversification in NK cells varies from individual to individual and is responsible for variable disease susceptibility against a variety of pathogens.

## NK cell plasticity in viral infections

During vaccination and viral infections, NK cells undergo phenotypic and functional changes and differentiation, resulting in the emergence of new types of NK cell subsets such as highly cytotoxic CD56^Dim^CD57^+^ mature, CD56^Dim^CD57^+^NKG2C^+^Fcϵr1g^+/–^ adaptive-like or CD56^-^CD57^+^ exhausted NK cells (4, 5). For instance, acute viral infections with viral pathogens such as influenza, dengue, West Nile, or SARS-CoV-2 virus increase the level of several cytokines such as IL-12, IL-18, and type I interferons in the host, which trigger activation and expansion of circulatory CD56^Bright^ and CD56^Dim^ NK cells and promote their homing to affected organs (4). On the other hand, chronic viral infections induce significant alterations in NK cell phenotype, composition, and distribution. In chronic human cytomegalovirus (HCMV) infection, adaptive-like NK cells (NKG2C^+^ or Fcϵr1g^–^, Fc epsilon receptor I gamma) appear, which exhibit enhanced antibody-dependent effector functions against infected cells and constitute up to 70% of total peripheral blood NK cells (6, 7). These HCMV-associated adaptive-like NK cells also expand during HIV (8), hantavirus (9), chikungunya virus (10), hepatitis virus (Malone et al.) and SARS-CoV-2 (11) infections in CMV seropositive individuals, where they impact disease outcome. Recently, severe COVID-19 disease has been found to be associated with adaptive-like NK cell expansion (11).

In this Research Topic, Brownlie et al. analyzed the expression of chemokine receptors CXCR3, CXCR6, and CCR5 on NK cells, which are involved in the recruitment of these cells to the lungs. In moderate COVID-19 and influenza patients, peripheral blood-derived CXCR3, CXCR6, or CCR5 positive NK cells were reduced and exhibited a stronger activated status, while in the bronchoalveolar lavage (BAL) fluid, there was an elevated concentration of the respective ligands of these chemokine receptors. These observations suggest the migration of activated NK cells from the periphery into lung tissue and their involvement in COVID-19 lung pathogenesis.

As NK cells exhibit strong activation and expansion during SARS-CoV-2 infection in the general population (11), the impact of SARS-CoV-2 infection on NK cell biology in pregnant women is not fully described. Carbonnel et al. showed that higher estradiol levels in pregnant women with SARS-CoV-2 infection suppress NK cells both phenotypically and functionally. This study hints that estradiol-induced NK cell suppression in SARS-CoV-2 infection may be a natural adaptation to ensure fetal survival by reducing immunopathology.

Viral vaccination has been shown to affect NK cell phenotype and function. Individuals with superior hemagglutination inhibition antibody titers after seasonal influenza vaccination showed higher frequencies of NKG2C^+^ adaptive-like NK cells (12). Additionally, vaccination-induced cytokine and chemokine responses, such as type I interferon, IL-12, IL-18, CCL2, and CCL4, have been reported for vaccines against influenza, Ebola, yellow fever, and hepatitis B viruses, and are involved in the activation, expansion, and trafficking of NK cells (5).

During chronic HIV and HCV infection, an exhausted CD56^–^ NK cell subset appears, which is associated with a higher viral load, and which comprises nearly half of the peripheral blood NK cells (13). These CD56^–^ NK cells were also observed to be expanded in CMV and Epstein-Barr virus (EBV) seropositive elderly healthy individuals, reflecting their immune risk profile (14). However, it is unclear how antiviral therapies for chronic viral infections impact NK cell subsets and what their effect on virus control is. Ivison et al., conducted a longitudinal study analyzing the NK cell receptor-ligand repertoire during long-term antiretroviral therapy (ART) in chronic HIV-1 infected patients. This study identified a less mature NK cell phenotype (CD16^+^CD56^Dim^CD57^–^LILRB1^–^NKG2C^–^), which was associated with lower HIV-1 cell-associated DNA. Further, surface expression of HLA-Bw6 on infected cells correlated with lower HIV-1 persistence. These findings uncover a link between the NK cell receptor and ligand repertoire and markers of HIV-1 persistence, suggesting a possible role for NK cells in regulating the latent HIV-1 reservoir. In another report, Sun et al. revealed the role of CD160 on NK cells in untreated HIV-infected individuals and its impact on viral control. They found that CD160 expression was highly reduced on NK cells of HIV-positive individuals, and this reduced expression of CD160 on NK cells correlated with disease progression. Mechanistically, the authors identified that CD160 positively regulates NK cell effector function by promoting the PI3K/AKT/mTOR pathway and glucose metabolism. Additionally, they showed that the higher TGF-β1 plasma levels induced by HIV infection inhibit NK cell CD160 expression, thereby promoting the disease. This indicates CD160 as a prognostic marker for infection with HIV.

Moreover, as summarised by Sun et al., NK cells undergo significant alterations in their receptor repertoire expression during HIV infection resulting in the emergence of different NK cell subpopulations, including protective, dysfunctional, and regulatory NK cells. The presence of a CD11b^+^ CD57^–^ CD161^+^ Siglec-7^+^ CD56^Dim^ CD16^+^ NK cell subpopulation has been suggested as a mean to discriminate elite HIV controllers from viremic non-controllers. Additionally, higher frequencies of CXCR5^+^ and Siglec-9^+^ CD56^Dim^ NK cells have been correlated with reduced viral load. Thus, these different NK cell subpopulations could play a role in spontaneous HIV infection control and may be useful for guiding the design of future antiviral therapies.

While several studies have examined how virus infections can change the NK cell receptor repertoire, it is important to note that the host genetic makeup of KIRs and HLAs genes also plays a significant role in regulating this repertoire (3). A study by Vollmers et al. identified that HIV-1-related changes in KIRs repertoire of NK cells are predetermined by host KIR2DL/HLA-C genotypes in viremic, untreated HIV-1^+^ individuals This suggests that KIR genotyping may be a predictive factor for NK cell-associated immune responses, and these findings can be utilized for improving NK cell-based immune therapies.

Besides performing antiviral functions and being involved in inflammation, NK cells can also indirectly promote virus persistence by presenting immunomodulating ligands such as PDL1 to corresponding inhibitory receptors on antiviral T cells (15), or by directly eliminating antiviral T cells (16–18), as shown in a murine lymphocytic choriomeningitis virus (LCMV) infection model. In humans, NK cells have been shown to inhibit the hepatitis B virus (HBV)-specific CD8^+^ T cell response (19). Further, highly differentiated NK cells were found to be negatively correlated with the generation of broadly neutralizing antibodies in HIV-1-infected individuals (20). Additionally, Liu et al. revealed a novel pathway of human antiviral CD8^+^ T cell regulation by NK cells in chronic HBV (CHB) infection. In particular, NK cells of CHB infected patients expressed higher levels of galectin-9 (Gal-9), a ligand for the inhibitory receptor Tim-3 expressed on effector CD8^+^ T cells, thereby inhibiting CD8^+^ T cell activation and the acquisition of effector function. These findings indicate that CHB infection-induced Gal-9 expression on NK cells promotes antiviral CD8^+^ T cell dysfunction, and they suggest that inhibition of this pathway could be a potential target for antiviral therapy.

Chronic HBV and HCV infections can lead to liver cirrhosis and hepatocellular carcinoma (HCC). NK cells play an important regulatory role in the development and progression of chronic HBV and HCV infection-induced HCC, which was comprehensively reviewed by Sajid et al.

## Impact of NK cell plasticity on NK cell diversity

Viral infections can have a substantial influence on NK cell diversity. During viral infections, NK cells can undergo activation, proliferation, and differentiation, resulting in significant changes in their phenotypic and functional properties. Studies have shown that short-term exposure to viruses such as HIV-1, West Nile Virus, or HBV results in the expression of previously un-expressed NK cell receptors such as NKG2C, CD57 and some KIRs, and hence increases NK cell diversity (21–23). On the other hand, chronic infections like HCMV or HIV/HCV can result in the clonal expansion of specific NK cell subsets such as adaptive-like and exhausted subsets to the detriment of others, thereby resulting in reduced NK cell repertoire diversity (21–23). Such reduction in NK cell repertoire diversity or inflexible NK cell repertoire due to too much diversification can impair the ability of NK cells to respond to new pathogens (23). Notably, changes in NK cell diversity caused by viral infections may persist even after the virus is cleared and may become permanent (21). The potential long-term consequences of these changes on the ability of the NK cell population to control heterologous infections remain unclear.

In conclusion, this collection of papers focusing on NK cell plasticity and diversity in viral infections advances our understanding of the multifaceted role of NK cells in antiviral immunity and virus immune evasion. It also highlights the importance of NK cells in influencing infectious disease outcomes in the host. Such a timely and updated view should trigger new thoughts for improving therapeutic strategies.

## Author contributions

All authors listed have made a substantial, direct, and intellectual contribution to the work and approved it for publication.

## References

[1] Freud AG Mundy-Bosse BL Yu J Caligiuri MA . The broad spectrum of human natural killer cell diversity. Immunity (2017) 47:820–33. doi: 10.1016/j.immuni.2017.10.008 PMC5728700 29166586

[2] Smith SL Kennedy PR Stacey KB Worboys JD Yarwood A Seo S . Diversity of peripheral blood human NK cells identified by single-cell RNA sequencing. Blood Adv (2020) 4:1388–406. doi: 10.1182/bloodadvances.2019000699 PMC7160259 32271902

[3] Horowitz A Strauss-Albee DM Leipold M Kubo J Nemat-Gorgani N Dogan OC . Genetic and environmental determinants of human NK cell diversity revealed by mass cytometry. Sci Transl Med (2013) 5:208ra145. doi: 10.1126/scitranslmed.3006702 PMC3918221 24154599

[4] Bjorkstrom NK Strunz B Ljunggren HG . Natural killer cells in antiviral immunity. Nat Rev Immunol (2022) 22:112–23. doi: 10.1038/s41577-021-00558-3 PMC8194386 34117484

[5] Goodier MR Riley EM . Regulation of the human NK cell compartment by pathogens and vaccines. Clin Transl Immunol (2021) 10:e1244. doi: 10.1002/cti2.1244 PMC7813579 33505682

[6] Lee J Zhang T Hwang I Kim A Nitschke L Kim M . Epigenetic modification and antibody-dependent expansion of memory-like NK cells in human cytomegalovirus-infected individuals. Immunity (2015) 42:431–42. doi: 10.1016/j.immuni.2015.02.013 PMC4537797 25786175

[7] Schlums H Cichocki F Tesi B Theorell J Beziat V Holmes TD . Cytomegalovirus infection drives adaptive epigenetic diversification of NK cells with altered signaling and effector function. Immunity (2015) 42:443–56. doi: 10.1016/j.immuni.2015.02.008 PMC4612277 25786176

[8] Guma M Cabrera C Erkizia I Bofill M Clotet B Ruiz L . Human cytomegalovirus infection is associated with increased proportions of NK cells that express the CD94/NKG2C receptor in aviremic HIV-1-positive patients. J Infect Dis (2006) 194:38–41. doi: 10.1086/504719 16741880

[9] Bjorkstrom NK Lindgren T Stoltz M Fauriat C Braun M Evander M . Rapid expansion and long-term persistence of elevated NK cell numbers in humans infected with hantavirus. J Exp Med (2011) 208:13–21. doi: 10.1084/jem.20100762 21173105 PMC3023129

[10] Petitdemange C Becquart P Wauquier N Beziat V Debre P Leroy EM . Unconventional repertoire profile is imprinted during acute chikungunya infection for natural killer cells polarization toward cytotoxicity. PloS Pathog (2011) 7:e1002268. doi: 10.1371/journal.ppat.1002268 21966274 PMC3178577

[11] Maucourant C Filipovic I Ponzetta A Aleman S Cornillet M Hertwig L . Natural killer cell immunotypes related to COVID-19 disease severity. Sci Immunol (2020) 5(50):eabd6832. doi: 10.1126/sciimmunol.abd6832 PMC7665314 32826343

[12] Riese P Trittel S Pathirana RD Klawonn F Cox RJ Guzman CA . Responsiveness to influenza vaccination correlates with NKG2C-expression on NK cells. Vaccines (Basel) (2020) 8(2):281. doi: 10.3390/vaccines8020281 32517137 PMC7349951

[13] Bjorkstrom NK Ljunggren HG Sandberg JK . CD56 negative NK cells: Origin, function, and role in chronic viral disease. Trends Immunol (2010) 31:401–6. doi: 10.1016/j.it.2010.08.003 20829113

[14] Muller-Durovic B Grahlert J Devine OP Akbar AN Hess C . CD56-negative NK cells with impaired effector function expand in CMV and EBV co-infected healthy donors with age. Aging (Albany NY) (2019) 11:724–40. doi: 10.18632/aging.101774 PMC6366961 30686790

[15] Zhou J Peng H Li K Qu K Wang B Wu Y . Liver-resident NK cells control antiviral activity of hepatic T cells *via* the PD-1-PD-L1 axis. Immunity (2019) 50:403–17 e4. doi: 10.1016/j.immuni.2018.12.024 30709740

[16] Duhan V Hamdan TA Xu HC Shinde P Bhat H Li F . NK cell-intrinsic FcepsilonRIgamma limits CD8+ T-cell expansion and thereby turns an acute into a chronic viral infection. PloS Pathog (2019) 15:e1007797. doi: 10.1371/journal.ppat.1007797 31220194 PMC6605677

[17] Waggoner SN Cornberg M Selin LK Welsh RM . Natural killer cells act as rheostats modulating antiviral T cells. Nature (2011) 481:394–8. doi: 10.1038/nature10624 PMC3539796 22101430

[18] Madera S Rapp M Firth MA Beilke JN Lanier LL Sun JC . Type I IFN promotes NK cell expansion during viral infection by protecting NK cells against fratricide. J Exp Med (2016) 213:225–33. doi: 10.1084/jem.20150712 PMC4749923 26755706

[19] Peppa D Gill US Reynolds G Easom NJ Pallett LJ Schurich A . Up-regulation of a death receptor renders antiviral T cells susceptible to NK cell-mediated deletion. J Exp Med (2013) 210:99–114. doi: 10.1084/jem.20121172 23254287 PMC3549717

[20] Bradley T Peppa D Pedroza-Pacheco I Li D Cain DW Henao R . RAB11FIP5 expression and altered natural killer cell function are associated with induction of HIV broadly neutralizing antibody responses. Cell (2018) 175:387–99 e17. doi: 10.1016/j.cell.2018.08.064 30270043 PMC6176872

[21] Strunz B Hengst J Deterding K Manns MP Cornberg M Ljunggren HG . Chronic hepatitis c virus infection irreversibly impacts human natural killer cell repertoire diversity. Nat Commun (2018) 9:2275. doi: 10.1038/s41467-018-04685-9 29891939 PMC5995831

[22] Strauss-Albee DM Fukuyama J Liang EC Yao Y Jarrell JA Drake AL . Human NK cell repertoire diversity reflects immune experience and correlates with viral susceptibility. Sci Transl Med (2015) 7:297ra115. doi: 10.1126/scitranslmed.aac5722 PMC4547537 26203083

[23] Wilk AJ Blish CA . Diversification of human NK cells: Lessons from deep profiling. J Leukoc Biol (2018) 103:629–41. doi: 10.1002/JLB.6RI0917-390R PMC6133712 29350874

